# Rethinking the Optimal Duration of Immune Checkpoint Inhibitors in Non-small Cell Lung Cancer Throughout the COVID-19 Pandemic

**DOI:** 10.3389/fonc.2020.00862

**Published:** 2020-05-12

**Authors:** Alex Friedlaender, Chul Kim, Alfredo Addeo

**Affiliations:** ^1^Department of Oncology, University Hospital of Geneva (HUG), Geneva, Switzerland; ^2^Georgetown Lombardi Comprehensive Cancer Center, Georgetown University, Washington, DC, United States

**Keywords:** NSCLC, COVID19, immune checkpoint inhibitors, duration, first line

## Abstract

Immune checkpoint inhibitors (ICPIs) have revolutionized the management and prognosis of fit patients with advanced non-small cell lung cancer (NSCLC). Recently, the publication of 5-year survival rates has cemented the role of ICPIs in NSCLC. An ongoing challenge is to determine the optimal treatment duration to find the balance between efficacy, toxicity and cost. From the onset of ICPI trials, different durations were used, ranging from treatment until progression or toxicity, to fixed durations of 2 years. Subsequently, exploratory analyses from a 1-year fixed duration trial failed to change practice. There are, to date, no adequately powered prospective trials addressing this important question. With today's severe acute respiratory syndrome coronavirus 2 (SARS-COV-2) pandemic, more than ever, the question resurfaces with added factors tilting the already shaky therapeutic balance. Here, we will discuss current data regarding ICPI treatment duration and incorporate this into the context of the ongoing pandemic. We conclude with a discussion of pragmatic approaches, should physicians be unable to continue standard therapy.

## Introduction

In March 2020, the World Health Organization declared a pandemic due to spread, number of cases and death caused by the severe acute respiratory syndrome coronavirus 2 (SARS-CoV-2) the case fatality rate seems to be about 3–4% but increases to 60.5% for critical cases (1). There are currently many trials ongoing, assessing the utility of antiviral and cytokine-directed therapies among patients with clinical manifestations of this virus. While no treatment has yet to prove its efficacy, standard management remains supportive, with organ support in intensive care for critically ill patients. Retrospective analyses of patients diagnosed with clinical manifestations of SARS-CoV-2 (2), the coronavirus 19 disease (COVID-19) found that cancer patients harbored a higher risk of infection than the general population (OR, 2.31; 95% CI, 1.89–3.02), regardless of whether they were on active treatment and that patients with lung cancer with COVID-19 are more prone to severe complications, including admission to the intensive care unit requiring invasive ventilation and death (3).

Immune checkpoint inhibitors (ICPIs) have revolutionized the management and prognosis of fit patients with advanced NSCLC (4, 5). An ongoing challenge is to determine the optimal treatment duration to find the balance between efficacy, toxicity, and cost. From the introduction of ICPI trials, there have been divergent treatment durations and there still has been no adequately powered, prospective trial comparing different treatment lengths (Table 1). The phase I Keynote 001 trial evaluated the efficacy of the anti-PD-1 inhibitor pembrolizumab in patients with advanced disease, both in treatment-naive and in subsequent lines, including a NSCLC cohort (6). Patients were treated until disease progression or unacceptable toxicity. The phase I CA209-003 (7) trial mirrored this design but with the anti-PD-1 inhibitor nivolumab, administered up to 2 years, and only in heavily pretreated patients (1–5 prior systemic regimens), including a NSCLC cohort. In the former, it was noted that patients still on treatment at 2 years had a 75% chance of being alive at 5 years. In both trials, the 5-year overall survival was 15% in the second- and subsequent-line setting, raising the question about optimal duration of ICPI therapy. Similarly, landmark practice changing phase III randomized clinical trials were designed with variable treatment durations, ranging from a predetermined maximum of 2 years of administration, to continuation of treatment until progression or unacceptable toxicity.

**Table 1:** Select trials in NSCLC illustrating the discrepancy in treatment durations.

**Trial**	**Checkpoint inhibitor**	**PD-L1 expression**	**Line**	**Duration**
CA-209-003	Nivolumab	All	Any	Up to 2 years
Keynote 001	Pembrolizumab	>1%	Any	Continuous
Keynote 010	Pembrolizumab	>1%	≥2	Up to 2 years
Checkmate 153	Nivolumab	All	≥2	1 year vs. continuous
OAK	Atezolizumab	All	≥2	Continuous
Checkmate 017/057	Nivolumab	All	≥2	Continuous

The optimal duration of therapy for ICPIs is unknown, but with today's SARS-COV-2 pandemic, more than ever, the question resurfaces with added factors tilting the already shaky therapeutic balance. Here, we will discuss current data regarding treatment duration and incorporate this into the context of the ongoing pandemic.

## What Do We Know About Treatment Durations?

The phase IIIB/IV CheckMate 153 trial is the first randomized trial to evaluate safety and efficacy of nivolumab treatment duration in NSCLC patients (8). It compared a fixed 1-year treatment regimen to a continuous one and exploratory analyses included the incidence of adverse events, progression-free survival (PFS) and OS. There was a significant PFS difference in favor of the continuous treatment arm [hazard ratio (HR) 0.42 (95% CI 0.25–0.71)], with an OS trend in the same direction HR 0.63 (0.33–1.20), both independently of the depth of response to therapy. It should be noted that this was an exploratory analysis among a small cohort of patients representing only a fraction (163/1,245) of the initial patient population, as only non-progressing patients could be randomized. Despite the limitations of potential trial biases and the immaturity of results, these data do not support pre-planned treatment interruption at 1 year.

The commonly used, initially arbitrary, 2-year treatment cut-off derives by certain study results. No direct comparison exists, but cross-trial analysis in similar patients and therapeutic constellations provides some insight into this difficult question. The CA209-003 cohort of advanced, previously treated NSCLC patients who received nivolumab for up to 2 years demonstrated a 3-year survival of 18% (9). The pooled analysis of the randomized CheckMate 017 and 057 trials, in the same therapeutic context, but with treatment until progression or toxicity, found a comparable 3-year survival of 17% in the nivolumab arm. Similarly, the 3-year survival among patients with continuous pembrolizumab in the Keynote 001 trial appears equivalent to that found in the 2-year fixed duration phase II/III Keynote 010 trial (6), at 21 and 23%, respectively. In the Keynote 010 trial, among patients who finished 2 years of treatment, 64% had an ongoing response at a median follow-up of 43.4 months. It should be noted, however, that only roughly 10% of patients completed the 2 years of ICPIs.

## The Impact of Immune-Related Adverse Events

In the OAK trial, assessing the efficacy of the anti-PD-L1 antibody, atezolizumab, until progression or unacceptable toxicity in previously treated advanced NSCLC, regardless of PD-L1 expression, patients, 28% of those in the experimental arm demonstrated long-term survival, defined as greater or equal to 24 months (10). Interestingly, 9% of long-term survivors had discontinued atezolizumab due to adverse events but 30% had immune-related adverse events (irAEs), compared to 16% in the non-long term survivor patient subset. What is interesting about these numbers is the potential causality. Could irAEs predict longer survival, or is the higher incidence of irAEs simply the result of longer time on treatment? In the Checkmate 153 trial, continuous nivolumab treatment resulted in grade 3 or higher immune-related adverse events in 8% of patients, compared to 4% in the 1-year fixed duration cohort, but very few new safety events took place after the first year of therapy (8). Among patients who received pembrolizumab in the Keynote 010 trial, the incidence of grade 3 or greater irAEs was 18% for those who completed 2 years of treatment, compared to 16% in the entire cohort. Furthermore, in Keynote 001, only three grade 3 adverse events were reported between years 3 and 5 of treatment. As such, the argument of increased toxicity is not greatly compelling as the decision factor to interrupt an ICPI treatment, at least for those who have been on long-term ICPI therapy.

As in the OAK trial, patients who developed irAEs were over-represented among long term survivors in the CA209-003 trial. In the former, only 56% of patients alive at 5 years completed the 2 years of therapy, and 25% discontinued nivolumab due to immune toxicity.

Regarding toxicity and efficacy, data have long supported the association between cutaneous irAEs and response to therapy in melanoma (11). In NSCLC, retrospective data supported the association between immune toxicity and favorable outcomes (12). Recently, it was prospectively demonstrated that both survival and response rates are correlated with the appearance of autoimmune toxicity among advanced NSCLC patients treated with ICPIs (13, 14). These patients maintain a favorable prognosis even after treatment interruption.

Among patients whose treatment was interrupted due to toxicity, in case of disease progression, data on ICPI rechallenge are encouraging. The largest series of ICPI rechallenge after an initial grade 2 or higher irAE showed that roughly half of patients did not incur either a relapse of immune-toxicity or a new episode of immune-toxicity, independently of the severity of the initial reaction (15). Hence, anti–PD-1 or anti–PD-L1 rechallenge appears to be feasible and safe. However, among patients who did not achieve an early objective response, only a minority had onset of objective responses following retreatment. Whether these responses may have occurred in absence of retreatment is unclear. Nevertheless, among patients without responses at the time the first serious irAE was detected, PFS and OS were improved with retreatment compared with those with treatment discontinued.

## Rechallenge After Preplanned Interruption

A second ICPI rechallenge scenario exists, namely in the context of progression after a fixed-duration ICPI treatment. The PACIFIC trial showed the survival benefit of consolidation durvalumab, an anti-PD-L1 antibody, administered for 1 year after definitive chemoradiotherapy for inoperable stage III NSCLC, regardless of PD-L1 expression (16). Of 40 patients in the experimental durvalumab arm, 20 received a subsequent ICPI, with a response rate of 0% (17). Data are scarce regarding the efficacy of ICPI rechallenge after fixed-term treatments in the metastatic setting, but this is a primordial question when considering treatment interruption.

In Checkmate 153, among the 43 patients who progressed after receiving 1 year of nivolumab, 34 (79%) were retreated with nivolumab. The median time between discontinuation and progression leading to retreatment was 10.3 months. Upon retreatment, the median duration of nivolumab was 3.8 months (range 0.1–17.5 months at time of database lock). In Keynote 010, after 2 years of pembrolizumab 25 (32%) of patients progressed and 14 (56%) were rechallenged with a second course of pembrolizumab. Nearly half of these patients remained sensitive to checkpoint inhibition and derived a clinical benefit from the therapy, with 43% partial responses (PRs) and 79% disease control rate (DCR).

Rechallenge in real life has been recently published (18). Data from 10,452 patients, treated with nivolumab, were collected. About half of the patients (53.4 %) received post nivolumab therapy lines, with 1,517 (29.6 %) of these receiving a second course of PD-1 inhibitors, either after a treatment-free interval, in the resumption group (*n* = 1127), or after chemotherapy, in the rechallenge group (*n* = 390). The OS was 15.0 months the resumption group and 18.4 for the rechallenge cohort. Irrespective of groups, the OS was longer in patients with an initial nivolumab treatment duration ≥3 months.

In the front-line setting, pembrolizumab administered to patients with PD-L1 expression ≥50% for up to 2 years leads to remarkable outcomes compared to chemotherapy. In the 3-year update, among 10 patients who progressed after this fixed-duration course of ICPIs, 7 (70%) responded to rechallenge (19). While all of these results are on small numbers of patients and long-term follow-up data on rechallenged patients are awaited, they remain encouraging to support the hypothesis of a retained sensitivity to rescue ICPIs.

## Financial Toxicity

With ICPI prices upwards of 10,000 dollars per 21 day course in the United states, and of 5,000 dollars in the United Kingdom, Switzerland and China, the financial burden on health care systems or individual patients cannot be ignored (20). As ICPIs have moved to the front-line setting and are administered as monotherapy or in combination with chemotherapy to all NSCLC patients without targetable mutations, with the exception of KRAS mutations (21, 22), cost has become increasingly important. This had led some healthcare systems to impose a fixed duration of therapy of 2 years, based on the above-mentioned arguments (23). For instance, in the United Kingdom, treatment is capped at 2 years, while in the United States and Switzerland, it remains at the physician's discretion.

## Can We Use Predictors of Long-Term Benefit to Determine the Duration of ICPI Treatment?

For the treatment of advanced melanoma, the type of radiographical response is used to guide the duration of ICPI therapy based on data suggesting complete response (CR) is associated with durable antitumor activity. In the Keynote-006 trial (24), early discontinuation of pembrolizumab was allowed if the patient achieved a CR and received the treatment for at least 6 months. Twenty three patients who met such criteria had a 24-month PFS rate of 86.4% (95% CI: 63.4–95.4), which was similar to that seen in those with CR who completed 2 years of pembrolizumab. Likewise, a real-world cohort study assessing outcomes after elective discontinuation of anti-PD-1 therapy in patients with melanoma demonstrated that complete responders who received treatment for at least 6 months had a low incidence of relapse (25). It is challenging to apply this treatment paradigm directly to lung cancer because complete response is rarely seen with ICPI therapy in lung cancer, reflecting different sensitivity to ICPI treatment between melanoma and lung cancer. In the CheckMate 153 trial, only 2 (3.6%) of 56 patients in the 1-year treatment group had a CR. Furthermore, CheckMate 153 showed that more than half of patients who achieved either a CR or PR with 1-year of nivolumab experienced a relapse within a year. Fluorodeoxyglucose (FDG)-positron emission tomography (PET) has been suggested to provide a better assessment of response to ICPI therapy than CT-based RECIST evaluation in melanoma patients (8). The role of FDG-PET in patients with lung cancer treated with an ICPI should be further explored.

Circulating tumor DNA (ctDNA) has been emerging as a useful tool to monitor response to various anticancer therapeutics including ICPIs. A recent study of ctDNA in long-term responders to PD-(L)1 blockade suggest that ctDNA testing can be used to predict the risk of eventual progression. In this study, 31 NSCLC patients with long-term benefit to PD-(L)1 blockade (defined as PFS ongoing > 1 year) were included (26, 27). The median duration of ICPI therapy was 20.4 months (range: 1.7–48.1 months). Surveillance ctDNA was obtained at a median of 26.7 months (range: 8.3–61.8 months) after initiation of therapy. Twenty five (92.6%) of 27 patients without detectable ctDNA have not progressed with median event-free survival since plasma collection of 17 months. In contrast, 4 patients whose ctDNA was detectable eventually progressed. The study is limited by the small number of patients and non-uniform timing of blood collection, but if validated in future studies, ctDNA analysis may offer a personalized approach to ICPI therapy.

Today, the only reliable positive predictive biomarker available for overall survival is PD-L1 expression (19). While it allows us to predict benefit, current data do not provide clues as to whether it could also predict durable responses with shorter treatment courses. Other possible biomarkers have been explored, among them tumor mutation burden (TMB), despite the challenges to measure it (28), has captured lots of attention given the initial positive results (checkmate 158, 227). Unfortunately it failed to predictive overall survival benefit (29, 30).

Other laboratory based biomarkers such as Lung Immune Prognostic Index combining neutrophil to leukocyte ratio (NLR) and lactate dehydrogenase (LDH) have been shown to have prognostic values in the setting of ICPI therapy, but their role in helping to determine the duration of ICPI therapy has not been well-defined (31).

## How Does SARS-COV-2 Impact Treatment Duration?

The above highlights the lack of consensus or clear clinical evidence supporting the duration of treatment. Enter the risk of COVID-19. We await robust data, though none is currently unavailable, on a potential link between immunotherapy and the incidence, or development of severe forms of this infection. However, there is an over-representation of cancer patients among those affected by COVID-19 and patients with lung cancer might represent up to 28% thereof (32). Furthermore, Garassino (33) recently presented the preliminary data of the TERAVOLT (Thoracic cancERs international coVid 19 cOLlaboraTion) registry for thoracic cancers at the AACR 2020 conference. For the first 200 patients in the registry, the median age was 68 years, 73.5% had stage IV disease and 75.5% had non-small cell lung cancer. The majority of patients were hospitalized (76%), and 33.3% of these patients died. Univariate analysis did not show an association with any specific cancer treatment and an increased risk of death. Multivariate analysis adjusted for the most important risk factors in the general population did not identify a risk profile for COVID-19 mortality in patients with thoracic cancer. Thus, while the therapy itself may not impact the risk of infection, the inherent fragility of these patients makes each trip and consultation a potentially perilous affair.

Certain recommendations have been elaborated to decrease the frequency of administration by doubling ICPI dosage (34, 35). However, not in every country health authorities allow the 4–6 weekly ICPI schedule. Furthermore, the half-life of anti PD-1 antibodies is 12–20 days, and there is a sustained 2 month or greater occupancy of >70% on circulating T-cells following infusion, independently of the dose administered, even after the drug is no longer detectable in blood (36). Based on these data and given the circumstances we would therefore suggest a pragmatic approach. As such, among patients in whom treatment must be continued, it would seem reasonable to allow a greater interval, after a single or double dose of PD-1 inhibitors, in order to protect fragile patients from unwarranted exposure to SARS-CoV-2 during this period.

In healthcare systems in which treatment duration remains at the discretion of the oncologist, we would err on the side of caution and stop at 2 years. Given the exploratory results of Checkmate 153 and the PFS benefit and OS trend favoring continuous treatment over a 1-year fixed course, we cannot reasonably suggest this as standard practice. Nonetheless, for a very fragile and comorbid patient in whom ICPI was previously initiated, a treatment interruption could be evaluated.

Data on efficacy of ICPI in patients with poor performance status (e.g., ECOG PS 2) are rather scant (37). Two trials have been recently published: PePs2 and the GOIRC-2018. The primary endpoints of the PePS2, which was a prospective phase II single-arm trial (38), were toxicity and durable clinical benefit, defined as disease stability or better at 6 months. A total of 60 patients with PS ECOG 2 were recruited among the 122 assessed. They received single-agent pembrolizumab, 24 patients in the first-line and 36 in subsequent lines. The treatment showed a good safety profile. The overall response rate was 27% (15/60 patients), with half of them in patients with PD-L1 >50% (47%, 7 patients). The authors rightfully reported OS data showing a median OS ranging from 8.1 to 14.6 months in PD-L1 <1 and >50%, respectively. They also suggested that their findings were numerically similar to what was observed in fit patients in the KEYNOTE-001, where 18% of patients achieved an objective response, with a median OS of 9.3 months. We would be more cautious as the group of patients assessed in the PePs2 was rather small.

The GOIRC-2018 is a retrospective study including 153 patients with PS ECOG 2 and PD-L1 >50% who were treated with front-line pembrolizumab (39). At a median follow-up of 18.2 months, median PFS and OS were 2.4 and 3.0 months, respectively. The final outcome was globally dismal but also strongly dependent on the reason conditioning the poor PS itself as patients with a PS 2 determined by comorbidities had significantly better outcomes compared with disease burden-induced PS 2. Therefore, the risk-benefit ratio of ICPI therapy in those with poor performance status (e.g., ECOG performance status 2) (5) is not as favorable as in medically fit patients and with the added risk of exposure to SARS-CoV-2 amidst the COVID-19 pandemic, a thoughtful discussion of potential risks and benefits associated with ICPI treatment needs to be held with those patients.

We repeat that data available at the moment are weak and scant and that if and when possible, all treatments should be continued as usual to offer optimal proven cancer care. We must ensure that disruption of cancer services won't cost more lives than the Covid-19 on its own.

## Data Availability Statement

The original contributions presented in the study are included in the article/supplementary materials, further inquiries can be directed to the corresponding author.

## Author Contributions

All authors listed have made a substantial, direct and intellectual contribution to the work, and approved it for publication.

## Conflict of Interest

AA has received compensation from Bristol-Myers Squibb, AstraZeneca, Merck Sharpe & Dohme, Takeda, Pfizer, Roche and Boehringer Ingelheim for participating on advisory boards. AF has received compensation from Roche, Pfizer, Astellas and Bristol-Myers Squibb for service as a consultant CK has received research grants (to institution) from AstraZeneca, BMS, Novartis, Regeneron, Tesaro, Karyopharm, Debiopharm, and Altor Bioscience, and has served on the advisory board of Novartis.
